# Salivary Cortisol Interactions in Search and Rescue Dogs and Their Handlers

**DOI:** 10.3390/ani10040595

**Published:** 2020-04-01

**Authors:** Justyna Wojtaś, Mirosław Karpiński, Piotr Czyżowski

**Affiliations:** Department of Animal Ethology and Wildlife Management, University of Life Sciences in Lublin, Akademicka 13, 20-950 Lublin, Poland; justyna.wojtas@up.lublin.pl (J.W.); piotr.czyzowski@up.lublin.pl (P.C.)

**Keywords:** search and rescue dog, canine, handler, salivary cortisol, hormonal interaction

## Abstract

**Simple Summary:**

Stress affects both people and animals every day. Working dogs are exposed to the same stressors as their handlers during work. Our research was conducted during search and rescue dog exams. The aim of the study was to investigate if handler stress during the exam affects his or her dog’s stress level. We observed a strong relationship of salivary cortisol between the dogs and their handlers, which was most prevalent in female dogs and female handlers.

**Abstract:**

Search and rescue dogs are an important link in the search for missing persons. The aim of the study was to assess exam stress in search and rescue dogs and their handlers. The study included 41 rescue teams taking exams of field and rubble specialties. The level of cortisol, which is the main glucocorticosteroid modulating stress reactions in humans and dogs, was analyzed. The biological material used to assess the hormone concentration was saliva collected in a non-invasive way. In total, 164 test samples were collected: two from the dog and two from the handler before and immediately after the exam. Rescue exams were shown to significantly increase salivary cortisol in both dogs and their handlers. Strong interactions between cortisol levels in human–dog teams were also found with a more pronounced effect in female dog–female handler dyads.

## 1. Introduction

Understanding the relationship between dogs and their owners has a significant impact on the quality of life of both the dog and the human. These interactions may also affect the efficiency of working dogs.

The dog has been accompanying humans for the longest time of all animals, [1]; hence, dog welfare has been the subject of many scientific studies. To date, stress levels have been assessed in shelter dogs [2], dogs participating in agility competitions [3], army dogs [4], and search and rescue (SAR) dogs [5,6]. It has been confirmed that humans and dogs react to the same stress stimuli in a similar way [7]. There have been reports of a relationship between the reactions to acute [8] and chronic [9] stress in humans and dogs. There are also increasing numbers of publications on the human–dog interactions and their effects on health [10] and hormone levels in both these species [11,12].

An effective attempt to assess dog–human behavior relationships was initiated in the 1970s by Ainsworth et al. The test they proposed has been successfully used to this day [13,14]. The behavioral and physiological symptoms of dog–human cooperation based on the level of hormonal indicators are described by Payne et al. [15]. In turn, the influence of various human personalities on dog’s attachment to man can be found in the work by Zilch-Mano et al. [16]. The use of dogs in dogotherapy and the increase in the importance of an assisting dog has contributed to the development of research on human–dog interactions to increase mutual benefits and maintain mutual well-being by, e.g., reducing stress, pain, and anxiety [17]. Appropriate selection of personality for a human–dog pair, their individual features, training techniques, and mutual relations between the guide and the animal influence the results of the working team and the final therapeutic success [18]. Performing personality tests before the exam is necessary because some temperament traits, e.g., bravery/courage or anxiety, may be related to the race or type of use through mutations in specific genes, resulting from human selection for specific phenotypic or utilitarian conditions [19]. The work of rescue dogs and their daily training should be treated as fun, which can be observed every day in homes where the owners play with a dog, e.g., with a ball [20].

The aim of the study was to assess the level of examination-related salivary cortisol level in search and rescue dogs and their handlers. The research hypothesis assumed the existence of interactions between the handler and the SAR dog manifested by an increasing salivary cortisol level in the dog together with an enhanced salivary cortisol level in the handlers. The hypothesis was verified by analyzing cortisol levels in saliva sampled from the dogs and their owners.

### 1.1. Stress and Cortisol

The hypothalamic–pituitary–adrenal (HPA) axis is one of the biological systems involved in stress response of the organism. In the presence of a stressor, the hypothalamus produces corticoliberin, i.e., a corticotropin-releasing hormone (CRH). It stimulates the pituitary gland to secrete adrenocorticotropic hormone (ACTH). An increase in its concentration triggers the release of glucocorticosteroids from adrenal glands. Secretion of these hormones is a basic indicator of the stress response. Cortisol is extensively used as a measure of HPA axis activity [21]. It is the main glucocorticosteroid representative and a good measure of stress in both humans [22] and dogs. This natural steroid hormone produced by the zona fasciculata of the adrenal cortex exerts a considerable effect on metabolism. It influences carbohydrate (increased glycemia), protein, fat, calcium, and water–electrolyte metabolism as well as the immune and hematopoietic systems [23]. It also exerts a mobilizing effect on the organism. Like adrenaline, cortisol is classified as a stress hormone. The major portion of cortisol (over 90%) circulating in blood is bound to transport proteins, mainly CBG (corticosteroid-binding globulin). Only 3%–5% of total plasma cortisol circulates in an unbound bioactive form, i.e., free cortisol [23,24].

Serum and plasma with heparin or EDTA are the most common biological materials used for determination of the total cortisol level. Cortisol concentrations are most frequently used for assessment of welfare and stress responses, especially in animals. Results of determination of blood cortisol levels may be biased, as blood sampling itself may be stressful both for animals [25,26] and for humans. Therefore, non-invasive collection of material is being increasingly used. The method contributes to reduction of the fear level in the patient [27]. Saliva, urine, feces, and hair (coat) are non-invasively sampled biological materials used for assessment of cortisol levels. Cortisol can be determined even in nails [28].

The saliva cortisol level is an extremely useful stress biomarker [29]. Free cortisol is a biologically active hormone fraction; hence, salivary cortisol measurements are regarded by some authors as a better method than measurements of its serum content [30]. Additionally, a strong positive correlation has been found between blood and saliva cortisol levels [25,31,32,33]. Fluctuations in serum cortisol are reflected in its concentration in saliva within less than 5 min [34,35].

Saliva sampling does not require involvement of medical personnel and a properly instructed person is able to collect this material. This is associated with great convenience and low costs. There are special tubes with cotton or synthetic chewing swabs available on the market for determination of cortisol in saliva. This mode of saliva collection is preferred and better than passive drooling into a tube [24,36]. Transport of saliva is not problematic, as the compounds contained therein exhibit high stability. Saliva collection is a completely non-invasive and painless procedure eliminating the stress associated with blood sampling [24]. This biological material can be collected from a dog in less than 4 min without an effect on the cortisol concentration [26].

### 1.2. Search and Rescue Dogs

Dogs are macrosomatic animals with a very well developed sense of smell. With this trait, they can be used in olfaction-based work. The dog’s sense of smell is useful in detection of cancer cells, explosives, drugs, or corpses. Currently, dogs are an important link in the broad-sense rescue work. Properly trained dogs are able to replace many people in their work. They are effective in extreme weather conditions and more reliable than modern equipment. Search and rescue dogs are trained to find live people. Depending on the area of work, these animals are classified as open-field, disaster, and avalanche rescue dogs. Dogs in the first group inspect large open areas, forests, and hard-to-reach spaces. Dogs in the second group are trained to locate live humans in rubble left by construction and natural disasters, while avalanche dogs are trained to scrutinize avalanche sites systematically. 

Search dogs in Poland are very well trained, and the skills of rescue teams are thoroughly verified during state examinations. After succeeding in a specialized rescue dog examination, the animal can be admitted to civil or uniformed rescue teams.

Search actions cause extreme physical and mental exhaustion in the dog. Open-space search requires very good physical status, because a SAR dog often has to explore a large variously shaped terrain. The animal often works with a large number of rescuers and other search dogs, which requires appropriate concentration. Sometimes, after finding the missing person, the dog must immediately start to look for another one, which also requires special training and mental attitude. Search in rubble requires caution and can be dangerous for the dog. The animal must work alone, as the dog handler cannot enter the disaster area for safety reasons, and has to move only along designated paths.

## 2. Materials and Methods 

The investigations were carried out during state examinations for SAR dogs in Poland. The permission for the dog study was obtained from the 2nd Local Ethics Committee for Animal Experiments in Lublin (Resolution no. 68/2015 of 30 June 2015). Additionally, the research was approved by the organizer of the examination and each rescue dog handler involved in the experiment (written consent).

### 2.1. Rescue Teams

The investigations involved 41 rescue teams, i.e., 41 rescue dogs and 41 handlers (18 males and 23 females) all aged 25–56 years. The participants did not report chronic diseases or permanent drug intake both by themselves and by the dog. They were not tobacco smokers and had not drunk coffee for at least 30 min before the test. The handlers were the owners of the dogs, and the animals lived with them at home on a daily basis. They were fed in accordance with the recommendations of the Regulation of the Interior Minister of December 13, 2012 on animals used in rescue operations. The study involved 17 Labrador Retrievers, 8 German Shepherds, 6 Border collies, 2 Belgian Malinois Shepherds, 2 Golden Retrievers, 1 Bernese Mountain Dog, 1 Chesapeake Bay Retriever, 1 German Spaniel, 1 Welsh Springer Spaniel, 1 English Springer Spaniel, and 1 mixed-breed dog. The examined dogs were mostly males (30 males vs. 11 females). Fifteen females and one male had been sterilized. The mean age of the dogs in the study population was 5 years (SD = 2.18, SE = 0.34). 

### 2.2. Examinations 

The experiment was carried out during Class 0 and I disaster and open-field search examinations. Each examination consisted of an assessment of the dog’s behavior within the human and a search trial. During the test of the dog’s behavior towards the human, the handler with the dog on a leash approaches the examiner who greets them, walks away at a distance of 10 m, and lies down on the ground. The handler unleashes and orders the dog to work. The animal should signal once finding the person in one of the possible ways, i.e., by barking, fetching a roll, or reporting. Next, the handler ties up the dog on a loose leash and walks away calmly. The examiner approaches the dog and unties the animal. He leads the dog to a different place and ties the animal up again. The dog should not show signs of aggression or fear; otherwise, the team is not allowed to proceed to the main part of the examination, i.e., the search, which consists of locating shams hidden in a given area. Detailed guidelines for such examinations in Poland are specified in the aforementioned Regulation. The study material was collected at the time between the assessment of the dog’s behavior in relation to the human and the search test, i.e., immediately before and after the test. In the study, the disaster search and open-field search examinations were taken by 23 and 18 rescue teams, respectively. In total, 28 teams passed the test, whereas 13 did not obtain a license. Due to the circadian rhythm of cortisol secretion in humans, the analyses were not carried out during night examinations. In turn, the day examinations began after the morning cortisol release peak [30]. No circadian rhythm of cortisol secretion has been detected in dogs [33,37,38].

### 2.3. Biological Material

Saliva was collected from both the dog and the handler before and immediately after the examination (approximately 30 min after collection of the first sample). Salivette Cortisol tubes (Sarstedt, Germany) equipped with synthetic swabs were used for collection. Supervised by the investigator, the dog handlers collected their biological material themselves, following the instructions provided by the tube manufacturer. Saliva was collected by chewing a cotton swab instead of passive drooling [24]. Dogs’ saliva was collected by rubbing the cotton swab on their lips and the inside surface of the cheeks. If necessary, saliva secretion was stimulated with the help of a treat given to the dog after the procedure. No swabs with citric acid were used for stimulation due to their possible effect on salivary cortisol levels [39]. 

### 2.4. Determination of Salivary Cortisol Concentrations

Collection of the biological material was not problematic for both the handlers and the dogs. In total, 164 samples were obtained for the analyses. Tubes with soaked swabs were centrifuged (3600 rpm, 10 min) within one hour post-collection, and the centrifuged saliva was frozen (−20 °C). After thawing, heating, and re-centrifugation of the biological material, the cortisol concentration was determined with the DRG Salivary Cortisol HS ELISA assay. The procedures followed the manufacturer’s instructions. Cortisol concentrations were expressed in ng/mL.

### 2.5. Statistical Analysis

The results were analyzed statistically using the Statistica 13.1 PL statistical package. The distribution of cortisol levels in the dog handlers did not deviate significantly from the normal distribution both before (Shapiro–Wilk test, W=0.97, p=0.343>0.05) and after (Shapiro–Wilk test, W=0.96, p=0.180>0.05)the examination. The distribution of cortisol levels in the dogs deviated significantly from the normal distribution both before (Shapiro–Wilk test, W=0.93, p=0.018<0.05) and after (Shapiro–Wilk test, W=0.93, p=0.013<0.05)the examination. For this reason, nonparametric tests were used to assess the significance of the differences. The significance of the differences in the handlers’ cortisol levels before and after the examination was analyzed using the dependent-sample *t*-test. A nonparametric Wilcoxon test was employed to analyze the increase in cortisol levels in the dogs during the examination. Spearman’s rank-order correlation coefficient was used to analyze the relationship between the cortisol levels in the dogs and the handlers. The relationships between cortisol levels in the handlers and dogs with reference to the sex were analyzed using Pearson’s linear correlation coefficient r. Statistical significance was assigned at the typical significance level of p≤0.05.

### 2.6. Methodological Limitations

Due to the objective difficulties in the creation of a zero group and the lack of established reference values for the salivary cortisol level in dogs, we compared our results to the results published by other authors. Teams come for the exams from all over the country, and the journey itself is stressful for dogs. Therefore, taking a control sample at the test site would be pointless. The control sample would have to be taken at home in a dog-neutral environment, and this was not possible. In addition, we are aware of the large inter-individual and intra-individual variability, and we know that many factors may affect salivary cortisol levels in dogs [21], which is why our test results should be interpreted with extreme caution.

It was not possible to supplement the methodology by measuring the heart rate in dogs using a heart rate monitor. The rules of the rubble exams allow the dog to be equipped only with a collar, which will break apart if it is caught on the elements of the rubble. These restrictions are introduced for security reasons.

The exam regulations do not allow the exam to be recorded using a camera. In addition, both the field exam and the rubble exam involve work in hard-to-reach places where it is impossible to observe the dog. The only element in the assessment of animal behavior was the dog’s behavior test in relation to man.

## 3. Results and Discussion

### 3.1. Salivary Cortisol Level in Dog Handlers

The basic descriptive statistics for salivary cortisol levels in the handlers before and after the examination are presented in Table 1.

Due to the large daily fluctuations in cortisol levels in humans, the reference values of cortisol levels in human saliva have a very wide range. As shown by Musiała et al. [23], the reference values are in the range of 1.89–10.37 ng/mL. The mean cortisol concentrations in handlers’ saliva assessed before the examination in the present study were within the reference values. In turn, the mean cortisol levels in the handlers after the examination exceeded the reference levels specified for this hormone [23,40]. The results obtained in the present study, both before and after the examination, were substantially higher than the values of salivary cortisol determined in a study of examination-related stress among students conducted by Ng et al. [41] or Takatsuji [42]. In both publications, the levels of stress hormone before the examination did not exceed 2 ng/mL. Rescue examinations are undoubtedly a stressful situation for dog handlers. Examination stress in rescue dog handlers was also studied by Lit et al. [43]. The authors reported a higher level of salivary cortisol before and after the examination than in the control group; however, it was lower than in the present study.

**Figure 1:**
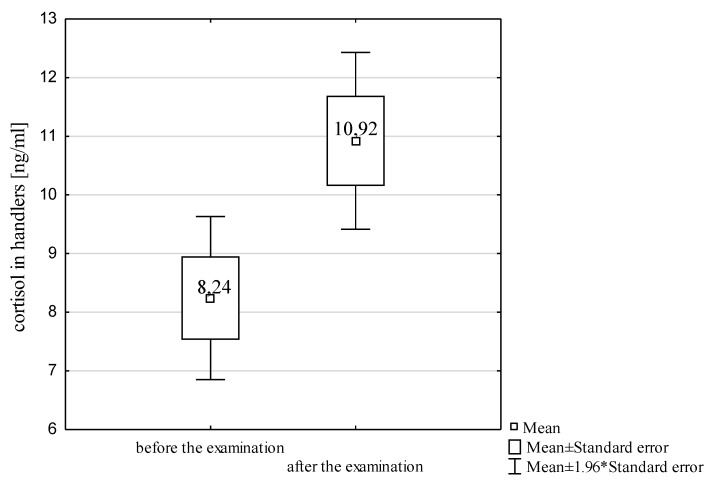
Box plot of the cortisol level in the dog handlers before and after the examination.

The level of cortisol was found to increase significantly in the handlers during the examination (dependent-sample *t*-test, t=−6.30, p<0.001; Figure 1). The examination is, therefore, a highly stressful factor, which is manifested by the release of the analyzed stress hormone throughout the examination in amounts exceeding reference values for humans [23,40].

### 3.2. Salivary Cortisol Level in SAR Dogs

The basic descriptive statistics for salivary cortisol levels in the dogs before and after the examination are presented in Table 2.

The mean cortisol level in the examined dogs was 4.2 ng/mL and 4.89 ng/mL before and after the examination, respectively. Both these values were higher than the mean levels of salivary cortisol in stress-unexposed dogs reported by Beerda et al. [44] (1.89 ng/mL), Colussi et al. [45] (3 ng/mL), Wenger-Riggenbach et al. [33](0.48 ng/mL), and Vincent and Michell [32](1.7 ng/mL). This implies that the examination is a stressful situation for SAR dogs, and the stress is manifested by cortisol release. A higher mean cortisol level in comparison with neutral conditions, with a lower value than that determined in the present study, was observed in therapy dogs before the therapeutic session (below 4 ng/mL) [46]. The therapeutic session is therefore a stressful situation for these dogs although not to such a high degree as in the case of SAR dogs. In turn, in comparison with the present results, a two-fold higher concentration of salivary cortisol, i.e., 8.7 ng/mL, was determined by Hekman et al. [47] in healthy dogs hospitalized before planned veterinary procedures.

**Figure 2:**
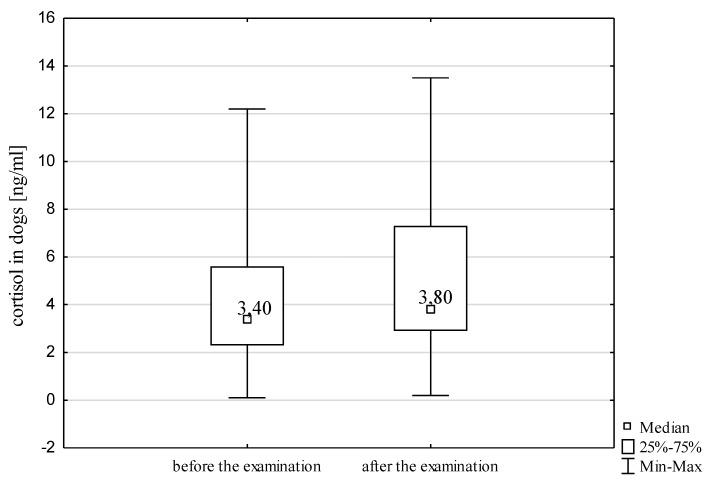
Box plot of the cortisol level in the dogs before and after the examination.

The level of cortisol during the examination was found to increase significantly in the SAR dogs (Wilcoxon matched-pair test, Z=2.47, p=0.014<0.05). This increase, however, was not as substantial as in the handlers. This is illustrated in Figure 2.

### 3.3. Correlation between Salivary Cortisol Level in the Handlers and Their Dogs

**Figure 3:**
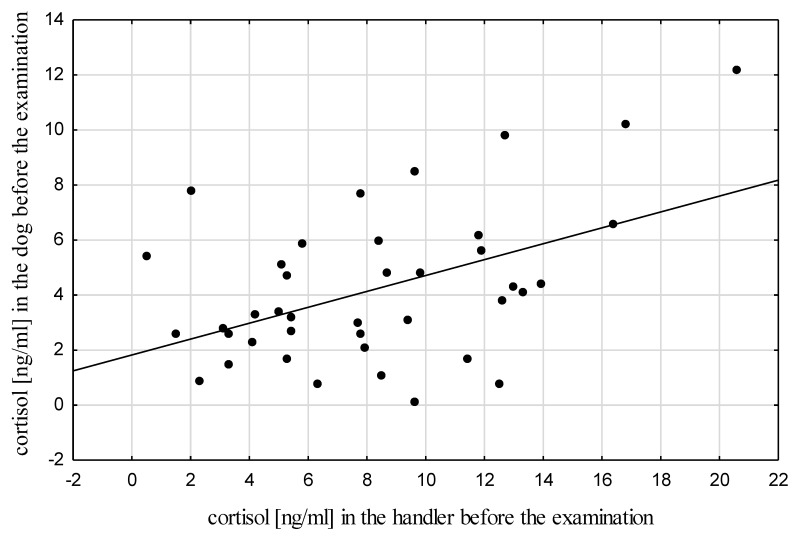
Scatter plot for the correlation between cortisol levels in the dogs and their handlers before the examination.

A significant positive correlation was found between the cortisol levels in the dogs and their handlers before the examination (Rs=0.34, p=0.032<0.05; Table 3, Figure 3), which may indicate that the dog senses the stress in the handler. A similar correlation was described by Buttner et al. [8] in a study conducted during agility competitions. As reported by other researchers, this correlation is found in not only acute but also chronic stress. Investigations of long-term stress in dogs and their owners assessed based on hair cortisol levels conducted by Sundman et al. [9] confirmed that dogs largely reflect their owners’ stress. An increase in cortisol concentrations in the owner was accompanied by an increase in the cortisol level in the dog. Schöberl et al. [48] reported unequivocally that a greater impact on the variability of cortisol levels in human–dog pairs is exerted by the human. Kotrschal et al. [49] found a significant impact of human personality (and thus susceptibility to stress factors) and behavior on the level of cortisol in dogs. Similarly, Schöberl et al. [50] concluded that the personality of the human and the relationship with the dog are the main determinants of stress reactions in the human–dog pair. Aggressive behavior of the owner towards the dog and punishment result in an increase in the cortisol level in the animal [51,52].

Dogs can perfectly recognize human emotions from body language and facial expression [53]. Mutual understanding in human–animal pairs is enhanced with time spent together doing the same tasks. SAR dogs and their handlers are occupied by training for many hours a week. As suggested by Meyer and Forkman [54], this may result in stronger hormonal dependence.

The results of the correlation analysis of the level of cortisol before the examination with reference to the sex of the handlers and dogs are presented in Table 4. There were strong positive correlations in the cortisol levels between the female dogs and the handlers. This correlation was found to be significant in the case of the female handlers (r=0.72, p=0.033<0.05),whereas a trend towards such a correlation was noted in the case of the men (r=0.81, p=0.096<0.1). Similarly, Sundman et al. [9] demonstrated a stronger relationship between the cortisol levels in dog owners and female dogs, in comparison with male dogs. As suggested by the authors, this can be explained by the greater emotional reactivity of females than males of many animal species and their different social roles.

### 3.4. Relationship between Exam Results and Stress in Handlers and Their Dogs

We analyzed the correlation between cortisol levels in dogs and handlers depending on the test result (positive or negative; Table 5).

A significant positive correlation of the cortisol level before the exam in the dogs and the handlers was observed in both passed and failed tests. However, this correlation in the case of the exam with a negative result is clearly stronger than the correlation of the cortisol level before the exam with a positive result.

Basic descriptive statistics for the cortisol level in the dogs and the handlers depending on the result of the exam and the cause of exam failure are presented in Table 6.

The mistakes that a dog can make include moving away from the helper three times without correct marking, with false marking, and the use of claws or teeth against the helper. An example of a guide’s mistake was to skip areas where the helper was hidden during the search.

In the observed sample of dogs that had made a mistake during the exam, the mean cortisol level (M = 5.41) was higher compared to the dogs that had passed the exam (M = 3.89). The cortisol level in the dogs in this group was strongly positively correlated with the cortisol level in their handlers. The cortisol level in handlers of dogs with a negative test result (M = 9.94) exceeded the mean cortisol level in handlers whose dogs passed the test (M = 8.31). Similarly, Lit et al. [43] observed elevated cortisol levels in handlers whose dogs failed the certification. It can be assumed that the dog’s mistake during the exam was associated with a high cortisol level, due to the strong significant positive correlation of cortisol levels in dogs and handlers. A similar relationship was described by Sümegi et al. [55]. They concluded that dogs of stressed owners are worse at solving problems and have poorer cognitive abilities.

The situation was completely different when the handler made a mistake. In this group, the mean cortisol level in the handlers was low (M = 3.92) compared to the mean cortisol level in handers with the positive exam result (M = 8.31). This difference cannot be considered significant in the population, but despite such a small number (n = 4), it can be classified as a tendency to a lower cortisol level in guides who made a mistake during the exam (*t*-test for independent tests, *t* = 1.78, p = 0.085 <0.1).The analysis of the cortisol level depending on the loss or win was carried out by Elias [56]. He observed a greater increase in cortisol levels compared to the baseline among the winners than the losers. Therefore, the cortisol values 10 and 35 min after the competition were higher for the winners than for the losers. Suay et al. [57] also observed that winners show higher cortisol levels than losers. In contrast, higher cortisol levels in competition losers than in winners were described by Gatti and De Palo [58], González-Bono et al. [59] and Gladue et al. [60].

We realize that the analysis of the results of examinations, and especially the causes of failing the exam, were carried out based on a small size of the sample (only 13 failed exams). Therefore, we treat this part of the analysis as preliminary research requiring continuation in the future.

### 3.5. Relationship between Exam Specialty and Stress Correlation in Handlers and Their Dogs 

We analyzed the correlation between salivary cortisol levels in the handlers and dogs by the type of exam. The table below contains the values of correlation coefficients only in groups determined by the guide’s sex, sex of the dog, and the moment of the exam in which the correlations proved to be significant.

A significant, very strong, positive correlation of the salivary cortisol level was observed before the exam in handlers and female dogs during the open-field exams (Table 7). In the same group before the exam on rubble, we observed a tendency towards a negative correlation between the salivary cortisol levels in the handlers and the female dogs. Zubedat et al. [61] examining dogs detecting explosives found that the higher the level of stress in a handler, the better the efficiency and effectiveness of the dog’s work. They suggested that, resultantly, less control of the stressed handler over the dog allows the dog to take control during the work. However, to be able to interpret the work of female dogs in the rubble in this way, it would be necessary to analyze a larger research group; hence, the present studycan be a preliminary study. In the studied population, only fivefemale dogs took the rubble exams. In the group of women, a significant strong positive correlation of the salivary cortisol level before the exam was observed in the handlers and their dogs during the fieldwork. It is worth noting that all statistically significant correlations between the salivary cortisol levels in the handlers and the dogs were found in the group of females. In terms of the specialty of the exam, these relationships were noted during the field examinations.

## 4. Conclusions

The handler–SAR dog teams analyzed in the study spend several hours per week of training. They go to rescue operations in various weather conditions and at different times of day and night. Common training, search actions, successes, and failures can produce a mutual hormonal relationship between the dog and the handler.In our study, we observed a significant relationship between the salivary cortisol levels in the search and rescue dogs and their handlers. In addition, we observed that this hormonal relationship was stronger for females and female dogs than for males and male dogs.

## Figures and Tables

**Table 1:** Basic descriptive statistics for salivary cortisol levels in the handlers before and after the examination.

Cortisol in Handlers (ng/mL)	n	Mean	St. dev.	Confidence	Confidence
		M	SD	−95%	95%
Before the examination	41	8.24	4.55	6.81	9.68
After the examination	41	10.92	4.93	9.37	12.48

**Table 2:** Basic descriptive statistics for salivary cortisol levels in the dogs before and after the examination.

Cortisol in Dogs (ng/mL)	n	Mean	St. dev.	Median	Q25	Q75
		M	SD	Me		
Before the examination	41	4.2	2.77	3.4	2.3	5.6
After the examination	41	4.89	3.19	3.8	2.9	7.3

**Table 3:** Spearman’s rank-order correlation coefficient r between cortisol levels in the dogs and their handlers.

Correlation between Cortisol Levels in the Dogs and Their Handlers	Rs	*p*
Before the examination	0.34	0.032 *
After the examination	0.18	0.406
Increase in the cortisol level	0.13	0.406

* *p* < 0.05.

**Table 4:** Pearson’s linear correlation coefficient r between cortisol levels in the dogs and their handlers with reference to the sex of the handlers and dogs.

Correlation between Cortisol Levels in the Dogs and Handlers before the Examination	Female Dogs (n = 11)			Male Dogs (n = 30)		
	n	r	p	n	r	p
Women (n = 18)	6	0.72	0.033 *	12	0.44	0.157
Men (n = 23)	5	0.81	0.096	18	0.29	0.245

* *p* < 0.05.

**Table 5:** Pearson’s linear correlation coefficient r between cortisol levels before the examination with reference to exam results.

Correlation between Cortisol Levels in the Dogs and Handlers	Exam Result					
before the examination	**Positive**			**Negative**		
	n	r	p	n	r	p
	28	0.38	0.046 *	13	0.73	0.005 *

* *p*< 0.05.

**Table 6:** Comparison of cortisol levels in handlers and dogs before the exam depending on the result of the exam and the cause of failure.

Before the Examination.	Cortisol Level (ng/mL)				Pearson’s Linear Correlation Coefficient	
	in Handler		in Dog			
	M	SD	M	SD	r	p
Failed exam	3.93	2.71	3.65	2.76	0.82	0.095
Handler mistake (n = 4)						
Failed exam	9.94	3.33	5.41	3.19	0.74	0.023*
Dog mistake (n = 9)						
Passed exam (n=28)	8.31	4.77	3.89	2.62	0.38	0.046 *

* *p* < 0.05.

**Table 7:** Pearson’s *r* correlation coefficients for the cortisol level before the exam in the groups: dog = female, handler = woman depending on the type of exam.

Cortisol Level before the Examination	Exam Specialty					
	Field			Disaster		
Studied group	n	r	p	n	r	p
Female dog	6	0.89	0.019 *	5	−0.84	0.073
Female handler	12	0.62	0.031 *	6	−0.44	0.385

* *p* < 0.05.
